# Between empathy and anger: healthcare workers’ perspectives on patient disengagement from antiretroviral treatment in Khayelitsha, South Africa - a qualitative study

**DOI:** 10.1186/s12875-022-01957-8

**Published:** 2023-01-26

**Authors:** Tsephiso Nhemachena, Carmen Späth, Kirsten D. Arendse, Keitumetse Lebelo, Nompumelelo Zokufa, Tali Cassidy, Katherine Whitehouse, Claire M. Keene, Alison Swartz

**Affiliations:** 1 School of Public Health and Family Medicine, University of Cape Town, Cape Town, South Africa; 2 Médecins Sans Frontières, Khayelitsha Project, Cape Town, South Africa; 3 Médecins Sans Frontières, Southern African Medical Unit, Cape Town, South Africa; 4 Health Systems Collaborative, Oxford Centre for Global Health Research, Nuffield Department of Medicine, University of Oxford, Oxford, UK; 5 Department of Psychosocial and Psychoanalytic Studies, University of Essex, Essex, England

**Keywords:** Disengagement, ART adherence, Healthcare workers, Perspectives, South Africa

## Abstract

**Background & objectives:**

The benefits of long-term adherence to antiretroviral therapy (ART) are countered by interruptions in care or *disengagement* from care. Healthcare workers (HCWs) play an important role in patient engagement and negative or authoritarian attitudes can drive patients to disengage. However, little is known about HCWs’ perspectives on disengagement. We explored HCWs’ perspectives on ART disengagement in Khayelitsha, a peri-urban area in South Africa with a high HIV burden.

**Method:**

Semi-structured interviews were conducted with 30 HCWs in a primary care HIV clinic to explore their perspectives of patients who disengage from ART. HCWs interviewed included clinical (doctors and nurses) and support staff (counsellors, social workers, data clerks, security guards, and occupational therapists). The interview guide asked HCWs about their experience working with patients who interrupt treatment and return to care. Transcripts were audio-recorded, transcribed, and analysed using an inductive thematic analysis approach.

**Results:**

Most participants were knowledgeable about the complexities of disengagement and barriers to sustaining engagement with ART, raising their concerns that disengagement poses a significant public health problem. Participants expressed empathy for patients who interrupted treatment, particularly when the challenges that led to their disengagement were considered reasonable by the HCWs. However, many also expressed feelings of anger and frustration towards these patients, partly because they reported an increase in workload as a result. Some staff, mainly those taking chronic medication themselves, perceived patients who disengage from ART as not taking adequate responsibility for their own health.

**Conclusion:**

Lifelong engagement with HIV care is influenced by many factors including disclosure, family support, and HCW interactions. Findings from this study show that HCWs had contradictory feelings towards disengaged patients, experiencing both empathy and anger. Understanding this could contribute to the development of more nuanced interventions to support staff and encourage true person-centred care, to improve patient outcomes.

**Supplementary Information:**

The online version contains supplementary material available at 10.1186/s12875-022-01957-8.

## Background

The notable progress made in curbing the HIV epidemic can be attributed to innovations in prevention and treatment, and the widespread rollout of access to healthcare services for people living with HIV (PLHIV) [1]. Consequently, there has been a global reduction of up to 60% in AIDS-related deaths since the peak in 2004 [2]. However, the epidemic has not been fully conquered as nearly 38 million people are living with HIV globally [2]. South Africa has the largest population of PLHIV (an estimated 7.5 million) and the biggest ART programme in the world [2]. Although 92% of PLHIV in South Africa are aware of their status, viral suppression may not be achieved among them due to difficulties with retention in HIV care and adherence to ART at individual and system levels [3].

Interruptions in care or disengagement have become an obstacle to the gains of ART and achieving The Joint United Nations Programme on HIV/AIDS (UNAIDS) 95–95-95 targets [2]. In South Africa, 71% of PLHIV are on treatment and 86% have a suppressed viral load [4]. In a study in the Western Cape, South Africa, 22.6% of patients on ART were shown to have disengaged from treatment within 2 years [5]. Poor adherence leads to several adverse consequences, including an increased likelihood of HIV transmission and the development of resistance to medication, resulting in people needing to be switched to regimens with a higher pill burden and less tolerable side effects [6, 7]. Non-adherence is also associated with a greater risk of progression to advanced HIV disease and mortality [6]. The consequences of disengagement from treatment for the individual result in adverse impacts on the public health system. Hospitalisation due to advanced HIV disease and opportunistic infections further adds to the burden of public health in resource-limited countries [4]. Studies have shown that a large proportion of people present with advanced HIV disease after disengaging from care [8–10]. Severe illness was shown as a strong motivation for patients to return to care [11, 12]. In a study investigating patients’ adherence based on their perception of disease severity, using the health belief model, it was found that patients who experienced more symptoms were more likely to seek medical assistance [13]. This highlights that patients who return to care after disengagement may require hospitalisation and additional support due to advanced HIV disease, and opportunistic infections, further adding to the burden on public health in resource-limited countries [6].

Studies show that numerous challenges contribute to disengagement from HIV treatment, including stigma, mental health issues, non-disclosure, and poor access to healthcare [14–16]. Following a period of disengagement, a major barrier to re-engagement in sub-Saharan Africa is the anticipated punitive treatment and negative attitudes by HCWs towards patients who return after interrupting care [17–19]. Moucheraud et al. note that HCWs serve as gatekeepers and that they impact patients’ HIV care experiences, yet most studies on disengagement are from the patient perspective [20]. Less is known about the perspective of HCWs. This limits our understanding of, and approach to optimising the management of disengagement.

Investigating HCWs’ perspectives of disengagement could guide the development of interventions to equip HCWs with better strategies for coping with disengagement. Additionally, the exploration of HCW perspectives could influence interventions that aim to reduce the frequency of disengagement and could address some of the barriers to re-engagement. To address this critical gap in knowledge, this study aimed to explore how HCWs perceive patients who disengage from ART in Khayelitsha, South Africa.

## Methods

### Study design and setting

This is a descriptive qualitative study using in-depth interviews (IDIs) with HCWs. The study was conducted at Ubuntu Clinic in Khayelitsha, a peri-urban, low-resourced area in the Western Cape Province, South Africa. Khayelitsha has a high HIV prevalence, compared to the Western Cape Province [21]. Ubuntu clinic is the biggest primary care clinic in Khayelitsha, with over 8000 PLHIV enrolled on ART [21]. The study was designed by Médecins Sans Frontières (MSF) to inform the design of an intervention, called the *Welcome Service*, to support people who return to care after ART interruption. The service uses a variety of tools including workshops to address HCWs’ negative attitudes and behaviours toward people who interrupt ART, and mentorship to optimise HCWs’ clinical and counselling skills to be more confident and equipped to manage ART interruption [22].

### Study participants and data collection

In line with the Centre for Disease Control and Prevention’s definition of ‘Healthcare Worker’, we considered all clinical, non-clinical, and support staff who contribute to the care of a patient to fall under this definition [23]. The HCWs interviewed for this study included doctors, nurses, counsellors, data clerks, security guards, and occupational therapists working in the Ubuntu HIV clinic. Purposive sampling was used for the selection of participants in the study. HCWs were eligible for inclusion if they had been working at Ubuntu Clinic for more than 3 months and were permanently based at the clinic. All the respondents were > 18 years and were able to provide informed consent.

Permission from the Ubuntu Clinic management and the line managers of the participants was obtained so that the HCWs could be relieved of their duties for interviews, provided it did not interfere with patient care. HCWs were approached individually and invited to participate in private, awarding HCWs an opportunity to decline without feeling pressured to participate. If they agreed to take part, an appointment would be set to conduct the interview.

Semi-structured interviews were used to collect data from the participants, lasting between 45 and 60 minutes. A semi-structured interview guide was designed, tested on MSF staff, and refined before being used to interview participants for this study. Interviews were conducted mostly in English by CK, NZ, and KW. Some interviewees were unable to express themselves fully in English; however, NZ was able to conduct these interviews in isiXhosa (a local South African language, of which NZ is a native speaker). If conducted in isiXhosa, translation to English was performed manually during transcription. The interview guide was not adapted during the study. Using the interview guide (see appendix A), HCWs were asked to share their experience of working with patients who interrupt treatment and how they feel when dealing with a patient who is returning to care. They were also asked about the reasons why patients disengage from treatment and what they thought about their reasons for disengagement.

Interviews were conducted in private rooms with one participant at a time. Before the interview, the researchers informed participants that they could refuse to answer any question that they felt uncomfortable about, could stop answering halfway through a question, withdraw their answer to a question either during or after the interview, and withdraw their participation completely either during or after the interview. Interviews were audio-recorded, and data collection continued until saturation was reached. In total, 30 participants were interviewed, and all interviews were transcribed and analysed.

### Data analysis

The qualitative analysis for this study was led by TN. Transcripts were analysed using an inductive thematic analysis approach, following Braun and Clarke’s stages of thematic data analysis [24]. NVivo 12 Pro, a qualitative data analysis software, was used for coding and analysis. Transcripts were imported into Nvivo and read by TN. After familiarisation with the data, meaningful text from the transcripts was highlighted. Text selections were used as a basis for generating broad themes, linked to the research questions, and based on participants’ explanations and understanding of disengagement. These initial themes and interpretations of the data were discussed at length with AS and CS and compared against an independently prepared coding framework by KW. In the discussion, thematic sub-sections were refined, and a set of clear sub-themes were identified. Transcripts were then revisited, and appropriate themes and sub-themes were assigned to text segments.

### Ethical considerations and informed consent

This study was approved for ethics by the Human Research Ethics Committee at The University of Cape Town (Ref: 542/2019) and the Médecins Sans Frontières Ethics Review Board (Ref: 1947). All participants gave written informed consent in English or isiXhosa for voluntary participation before being interviewed and were not given any compensation. No identifying participant information was used in written outputs, and respondents were anonymised using an identifier. All methods were performed following the Declaration of Helsinki.

### Findings

#### Demographic characteristics

Demographic characteristics presented in Table 1 show that the study participants comprised 30 HCWs, most of whom were support staff. Participants’ ages ranged from 25 years to 65 years with most being between 25 and 34 years. There was a balance between those who had extensive experience working in the HIV field and those who had recently entered the field. Notably, 27% had been working in the HIV field for less than a year, and 20% for more than 16 years. With regards to time working at Ubuntu clinic, 10 (33%) of the participants had been working at Ubuntu clinic for less than a year and two for more than 16 years. Most participants (60%) were employed by the Department of Health and 40% were employed by non-governmental organisations.

**Table 1:** Demographic characteristics of study participants

Participants	Total n (%)
*Gender*	
Female	21(70)
Male	9 (30)
*Age categories (years)*	
25–34	11 (37)
35–44	9 (30)
45–54	7 (23)
55+	2 (7)
Missing information	1 (3)
*Occupation*	
Support staff (management, clerks, pharmacists, data capturers, security officers, counsellors, facilitators, social workers, occupational therapists)	20 (67)
Clinicians (doctors, nurses)	10 (33)
*Employed by*	
Department of Health (DoH)	18 (60)
Non-governmental organisation (NGO)	12 (40)
*Time in the HIV field (years)*	
< 1	8 (27)
1–5	7 (23)
6–10	4 (13)
> 10	11 (37)
*Time at Ubuntu clinic (years)*	
< 1	10 (33)
1–5	7 (23)
6–10	7 (23)
> 10	6 (20)

#### Themes

Table 2 shows the themes that emerged from the data. Three prominent themes emerged (1) Disengagement from HIV care is a public health problem that needs attention, (2) HCWs express empathy and understanding for patients who disengage, (3) HCWs express anger and frustration toward patients who disengage. These broader themes are further divided into sub-themes presented fully below.

**Table 2:** Main themes identified in the analysis

Main themes	Subthemes
1. Disengagement is a public health problem	
2. HCWs express empathy and understanding for patient disengagement from care	• Mental health challenges
	• Non-disclosure leads to disengagement and the dilemma of disclosing the HIV status
	• Concern for patients who disengage from treatment
3. HCWs expressing anger and frustration	• Patients who disengage from treatment do not take responsibility for their health
	• Patients’ reasons for disengaging from treatment are unjustifiable

#### Theme 1: disengagement is a public health problem

When the study participants were asked about patient disengagement from ART, most acknowledged that disengagement is a significant public health concern. Participants outlined several adverse effects of disengagement.

*“It’s a problem because a person will start all over and get sick and now hospitals have to [be] full because of this person who just decided to stop taking their treatment whereas they had a chance to take their treatment, so they don’t get sick”.* (Support staff, age 38).

Here, the participant explained that when the patient returns to care after a period of disengagement, they will require hospitalisation, burdening the healthcare system. The participant also described how disengagement impacts the individual as it is a threat to the person’s health. Another participant added that disengagement results in ART resistance that impacts one’s health.

*“It is a problem because like you know, like the virus becomes resistant to medication, so which is posing danger to the person who is not taking the medication”.* (Clinician, age 49).

Most of the participants described disengagement from treatment as a “problem”. A participant reported that the advent of ART seemed to be the solution to HIV but frequent disengagement from treatment was becoming a challenge to the treatment process.

*“I think it’s a big problem having worked in ARVs for a long time, it feels like we’ve hit like a second wave of the epidemic. Like initially, we had very sick people coming in, but nobody had been on ARVs before so you, we were picking up the patients who were the weakest and getting them on treatments. … but now there [are] people who’ve been on ARVs before and stopped so it makes their treatment more complicated”.* (Clinician, age 42).

The participants saw disengagement from treatment as a significant and growing public health problem.

#### Theme 2: understanding disengagement: HCWs express empathy

The participants showed an understanding of the multiple factors that influence engagement. They highlighted some reasons for disengagement that were justifiable from their perspectives. Their responses showed that they have some empathy towards patients as they acknowledged that patients may disengage due to mental health challenges and non-disclosure. Moreover, the participants showed concern for patients who disengage from treatment. The following section presents factors that lead to disengagement as noted by HCWs.

#### Mental health challenges

Most of the participants stated that mental health challenges affect patients’ ability to make decisions and contribute immensely to disengagement from treatment.

*“Most of the patients that are having mental illness default their ARVs because of their poor insight or their poor judgment…… the patient comes, let’s say he’s HIV positive on ARVs and then develops mental illness, and then because mental illness affects their judgment and their insight when they are psychotic and in a psychotic state, then they can’t reason, they just do things that are very weird and things that are very odd to people”.* (Clinician, age 49).

The participants drew an association between mental illness symptoms (lack of insight, poor judgment) and adherence. They noted that mental health altered patients’ thought processes and decision-making, which could contribute to disengagement. The participants perceived mental illness to contribute to adherence behaviour and noted that disengagement is not a choice, but a behaviour that is influenced by the patient’s mental health among many other challenges.

*“Some are not mentally okay, maybe that could also be, that could, I could say maybe it’s not a normal behaviour because they also go for depression; some they have like mental illnesses…”.* (Support staff, age 26).

The participants reported that disengagement from treatment reflected what the patients experienced in their lives. The participants stated that when they came across patients who were depressed, going through rough patches, or when lost hope in life, they were likely to disengage from treatment.

“*…because she was struggling with her compliance on ARVs and TB treatment, we referred the patient for an assessment for depression and the patient was depressed because there was a lot of things that happened. So, we started addressing those, she was seen by the psychologist and we started her on antidepressants.”.* (Clinician, age 52).

HCWs associated mental health challenges and disengagement from treatment and described the different ways in which this occurred. The participants noted that for some patients, addressing the mental health challenges in turn addresses disengagement.

#### Non-disclosure leads to disengagement and the dilemma of disclosing the HIV status

Non-disclosure was emphasised as one of the major reasons for disengagement.

*“I see a lot of patients who are not taking treatment well because of non-disclosure so they haven’t told anyone at home”* (Clinician, age 42).

HCWs shared their perspective on how non-disclosure contributed to disengagement. One participant imagined that in cases where the family and partner were unaware of their HIV status, one was less likely to take treatment in front of their family, resulting in disengagement.

“*I think it’s difficult disclosing and, if I haven’t disclosed to my partner but every night at eight o’clock, we sit and have supper and I must take my treatment, you know?”* (Clinician, age 52).

The participants highlighted that disclosure was not easy, yet it was important, especially to family and partners as patients spend a lot of time with these people. Hiding their status from these significant people made it difficult for patients to take their treatment. The participants showed empathy for patients who found disclosing difficult because there were some fears that patients had regarding disclosing their status which included the fear of being misunderstood. Although HCWs understood these fears and concerns, they emphasised the importance of disclosing to partners and family as they can provide support and help to ensure treatment adherence.

*“I strongly believe if a person discloses to a person, it will be easy for that person even to take the medication because there is a support. Disclosing can be hard because your family may not understand”* (Support staff, age 57).

The participants noted there was that risk of not being understood but one could also get support after disclosing. They noted that it was important to know to whom they disclose, as it would influence whether they got support or were judged.

*“I am not saying people should just talk about it if they are not yet comfortable, but they should not hide it from everyone, such as their families. I don’t think all your family members can judge you. You can even speak to your parent, privately, so you can get support in that way.”* (Support staff, age 38).

Disclosure might cause a person to be judged, misunderstood, and they might lose loved ones following disclosure.

*“So, sometimes they say it’s the reason they can’t disclose because the guy now is going to dump that person because of the HIV status”* (Support staff, age 42).

HCWs acknowledged that disclosure was a challenge, it was not easy, therefore, showing empathy for patients. They described how disclosure can result in one getting support or in other instances being discriminated against and stigmatised. HCWs understood the complexities of disclosure.

#### Concern for patients who disengage from treatment

The participants reported being worried about patients when they disengaged from treatment because they were aware of the adverse consequences of disengagement. They showed concern for disengaged patients, and they emphasised that they were willing to help them in ways that they could.

*“I get worried and concerned. I want to know the reasons so that we can tackle whatever challenges she has to overcome those challenges so that the patient can be able to take treatment”.* (Support staff, 46).

The participant with the quote above expressed the need for insight into the reasons for disengagement. The participant expressed concern for patients and reflected on their role in being able to help the patient so that they could tackle the challenge of disengagement.

*“I feel sorry for them because I see dangers in that because there is a possibility that one can lose her life if she doesn’t take her medication, well, especially the ARVs”.* (Clinicians, age 49).

*“It makes me feel sad, especially the young ones…., like joh! She’s still young to have this viral load”.* (Support staff, age 28).

These quotes highlight that some participants had sympathy for patients, and they expressed worry for these patients and their well-being.

#### Theme 3: HCWs expressing anger and frustration

Participant responses highlight the anger and frustration HCWs may have towards patients who disengage from care.

#### Frustration and anger

Most of the participants expressed frustration and anger towards patients who disengage from treatment. They noted that they did not understand why patients discontinued their treatment when it is lifesaving. In that light, the participants communicated that when patients did not take treatment, they were choosing not to save their own lives.

*“…that’s what frustrating me, and then somebody dies because of HIV. That I…I…I…I fail to understand, why they should die because they have treatment already. It’s only the people who do not know, who did not know about this HIV, or they did never started tablets or treatment, they never tested for HIV; then I would understand, but for somebody who started treatment and then they decided to leave and then they are sick like that and then they die, that’s what frustrated me”.* (Clinician, age 64).

*“…for example, why didn’t you take your ARVs; I don’t have any reason. Now you’re like, well you just… I just want to strangle her... because I don’t understand, I don’t understand how come the person will just disengage their treatment for no reason”.* (Support staff, age 42).

*“…you counsel them till you’re blue in the face and they don’t change. And then that, it gets frustrating in the end because you’ve got someone who’s sick where they wouldn’t be if they just took their treatment… it’s time-consuming. The fact that now when they come to the clinic, for starters they must come every month because their viral load now is high; they must attend a ROTF*^1^*counselling sessions every morning before they go to [the] pharmacy before they’re being seen by the sister or a doctor they must go to a group counselling the ROTF counselling and they will become now it will be seen and then so it’s time-consuming they end up going home late and they get a monthly appointment so every month they must be here”.* (Clinician, age 42).

The participants noted that working with patients who disengage from treatment was time-consuming because they needed special attention which lengthened the process. HCWs were tired and worn out as a result; most of the participants reported that patients who disengage from care frustrated them because they increase the workload when they were already overwhelmed.

*“They’re making me angry. I get angry. I want to slap them. Joh, joh, I get angry when someone defaults treatment”.* (Support staff, age 33).

*“I think the anger could be for you know, with the patient sometimes because you get a sense this patient just doesn’t care”.* (Clinician, age 52).

The participants used negative language that described their anger and frustration towards patients who disengage from treatment. The expression “joh, joh” adds emphasis to the extent of their frustration.

#### Patients who disengage from treatment do not take responsibility for their health

Most of the study participants alluded to the notion that when patients disengaged from treatment it means that they did not take responsibility for their health and did not prioritise their health. Most of the participants emphasised that for patients to remain in treatment it was the responsibility of both the HCWs and the patient. Moreover, while HCWs were doing their part, patients tended not to take responsibility for their health.

*“If the nurse says or if the doctor says come back on the 8* ^*th*^ *of March, make means to come back on the 8*^*th*^ *of March because this is your health, and you only have one life honestly…. It’s honestly your responsibility, your health honestly. Your health is your responsibility and then the clinicians and the doctors are there to help you; are there to support you; they’re there for anything you want to ask and want to know”*. (Support staff, age 26).

Some of the participants stated that all they can do as HCWs is to support patients so that they can adhere to their treatment, and it was the responsibility of the patient to ensure that they stay engaged in care.

*“They don’t take their responsibility, because I don’t see the reason for patients to just drop their medication without consulting with [a] doctor”.* (Support staff, age 38).

*“…but the patient also has a responsibility and it’s not my responsibility as a healthcare worker to go and tell your boss that you must come to the clinic every month because now you have this disease”.* (Clinician, age 49).

The above quotes highlight that the HCWs believed that it was ultimately up to the patient to adhere to their treatment and responsibility for their health. The study participants highlighted that there was only so much that they could offer to the patients, but the patients needed to take ownership of their lives and prioritise their health over anything else.

#### Patients’ reasons for disengaging from treatment are unjustifiable

When the participants were asked about their experience with patients who stopped taking their HIV treatment and later return to care, most noted that these patients gave excuses for disengaging from treatment.

*“Something like they went to Eastern Cape and then they didn’t, that is not a good reason to me…If they are going to and their appointments are not far from coming back and then they are going away, they must come and report so that we give them referral letters, and then they can be sorted there”* (Clinician, age 64).

HCWs think disengagement is unjustifiable, particularly as many have personal experience of navigating the challenges of taking long-term treatment themselves. Surprisingly, most participants who reported taking chronic medication themselves were the ones who showed less understanding for patients who interrupted treatment. They spoke about adherence in a personal way, highlighting that if they were adhering to treatment then the patients could also do the same. They compared ART medication and their chronic medication where they highlighted that it is not difficult to take pills every day.

“*I’m diabetic, I’m taking mine twice a day, I’ve never really forgotten because I’m like I have to take it. So, for me, it’s just an excuse... mostly excuses. …no, not at all there’s no excuse not to take it”* (Support staff, age 28).

*“I don’t see why it should be fatigue from one tablet. It could be fatigued from maybe more than one tablet… I think it’s very traumatic when you are taking more than one tablet”.* (Clinician, age 49).

The above quotes from study participants highlight that the participants regarded some reasons for disengaging from treatment as unjustifiable. Some of these reasons they stated were traveling to the Eastern Cape, pill burden, and treatment fatigue.

## Discussion

This study shows that HCWs experience internal conflict as they grapple with contradictory feelings of empathy and anger towards patients who disengage from care. These findings show that HCWs understand the complexities of treatment adherence and that they recognise patients are faced with difficult circumstances that lead them to disengage. HCWs acknowledged that disengagement is a public health problem and showed concern for patients who interrupt care. On the other hand, there is a strong opinion that patients need to take responsibility for their health. The findings of this study show that the anger and frustration HCWs feel toward patients who disengage stems from the belief that these patients are not taking responsibility for their health.

The HCWs perceived themselves as having some responsibility in ensuring patients adhere and that they were willing to do what was in their capacity to support patients to stay engaged. This is in keeping with findings from a study that explored provider opinions about responsibility for medication adherence, where HCWs felt it was their responsibility to educate a patient and give them the proper treatment; after doing this, it became the responsibility of the patient to take medication as prescribed [25]. In our study, HCWs showed an understanding that disengagement is a problem that needs to be addressed, in line with previous studies of adherence where disengagement is regarded as a public health problem [3, 26]. Knowing that HCWs see the seriousness of the problem and acknowledge their responsibility is encouraging and could translate into HCWs interest in being part of the solution. HCWs can undertake skills training so that they are better-equipped to assist patients who disengage from treatment [27].

HCWs expressed empathy when they perceived disengagement as out of patients’ control, for example, if owing to mental health challenges. Between 20 and 60% of HIV-positive adults suffer from some form of mental illness, with challenges such as depression, and other negative emotions affecting their disposition and motivation to access and adhere to treatment [26, 28, 29]. Additionally, these findings are consistent with evidence from a meta-analysis that looked at the association of depression and non-adherence which established that depression is a risk factor for non-adherence to treatment [29]. Since patient-focused studies have also indicated that mental health challenges are a contributor to disengagement there are implications for interventions [30, 31]. While psychological services available at public clinics are scarce, especially in low-resource settings, there is a need for additional psychosocial support for patients who disengage [32, 33]. Task-shifting in mental health has been found to be a feasible and effective strategy to rollout in resource-limited countries [34]. Therefore, task shifting could also be employed by training more HCWs to provide basic mental health support in the form of counselling for patients who interrupt treatment.

The findings of this study show that HCWs believed that non-disclosure could be a barrier to social support as they fear disclosure could result in stigma and abandonment. This echoes a study that indicated that non-disclosure is intertwined with stigma and poor social support [35]. Notably, the fear of stigma may prevent patients from confiding in others, leading to a lack of emotional support [35]. Understanding the impact that non-disclosure can have on adherence could help inform interventions to improve engagement. Support from HCWs and counsellors may assist patients with disclosure and equip patients with skills to disclose their status [36, 37]. A recommendation from one study was to equip HCWs with tools to support disclosure through facilitated discussions between patients and their families or partners, and to develop action plans to involve patients’ social support networks in their care plan [37]. PLHIV hesitate to disclose their status due to fear of HIV-related stigma, interventions tackling stigma at different levels from family to community, could help patients feel more comfortable in disclosing [36].

In this study, there were reasons HCWs considered justifiable for disengagement, such as mental health and challenges with disclosure, using their standard of justification. This reflects mismatched priorities between the patient and provider, where HCWs feel health should be the patients’ primary priority. Research has shown that patient-centred care promotes treatment adherence and leads to improved health outcomes [38]. There is a push to make care more person-centred, which acknowledges people holistically and recognises that health is one of many competing priorities that patients may experience [38–40]. Our study supports the need to introduce the person-centred approach to improve treatment adherence.

It is apparent that while HCWs understand the complexities of adherence, they have contradictory feelings of anger and empathy toward patients who disengage. Our findings demonstrate that HCWs’ perceptions of disengagement such as, patients should be responsible for their own health or they over-burden the health system when they disengage, all contribute to the negative attitudes and behaviours that they may portray towards people who disengage. This also contributes to their feelings of anger and frustration. Drawing from the cognitive behavioural theory (CBT), cognition processes of individuals, which include assumptions, judgments, appraisals, meanings attached, and assumptions, play a significant role in developing and maintaining emotional and behavioural responses to scenarios [39]. HCWs’ impressions and opinions of adherence determinants are important as they shape patient interaction and clinical care recommendations, influencing the success of adherence interventions [20, 41].

Literature shows that HCWs may treat patients who disengage harshly and mistreat them even when they want to reengage [42, 43]. In some cases, patients report abuse by clinic staff and in some cases HCWs punish patients by refusing to see them, making them come back the next day, seeing them last, or shouting at them [42]. The source of this behaviour could result from HCWs’ negative perception of patients who disengage, which may stem from the mismatched patient-provider prioritisation of health as well as the overwhelming and under-resourced work environment that makes it challenging for HCWs to provide these patients with optimal care, particularly as they often have increased medical and psychosocial issues. There is a need for interventions to address the negative feelings that HCWs might have towards patients who interrupt treatment, acknowledging their feelings of both anger and empathy in designing that approach. The Actual event, Belief, and Consequences (ABC) model, influenced by CBT, notes the first step to addressing HCWs’ negative interactions is to recognise the source of the feelings, prejudices, biases, and negative thoughts [44]. According to the ABC model, understanding and managing stressful reactions is of paramount importance to attain control over automatic, irrational thoughts and substitute them with rational, flexible interpretations that encourage well-being and productivity [44]. Therefore, it may be beneficial for interventions to cultivate an understanding of patients holistically so that certain generalisations do not translate into how HCWs engage with patients. Notably, addressing HCWs’ feelings of anger and frustration and promoting empathy toward patients could influence patients’ engagement and long-term retention in care [41].

In addition, HCWs themselves could be offered psychosocial support to deal with their feelings. Vesel et al. noted that the provision of coping and stress management techniques for HCWs helps them to persevere within difficult environments and this could potentially impact health service delivery and quality of care [45]. HCWs have difficulties in grappling with certain feelings of anger and frustration because of HIV patient disengagement, but we cannot forget the broader context of the system in which they function. They work in an overstretched health system with staff shortages, poor service delivery with inadequate and unaccountable managerial structures, fragmented services, financial or cash-flow problems, and little to no emotional support, all of which might contribute to their anger and frustration [35, 46]. Psychological support could be integrated into training programmes for HCWs to equip them with stress management skills. In a systematic review, it was shown that the practice of mental and physical relaxation activities led to a 23% reduction in stress levels compared to no intervention [47]. Other forms of psychosocial support for HCWs could include having dedicated spaces where health facility staff can debrief [48]. A study with doctors showed that debriefing sessions provided emotional and social support that in turn reduced burnout among participants [48]. Concrete supervision and support for HCWs could be a form of psychosocial support for health facility staff [48].

In essence, the findings of this study are valuable as they could be translated into psychosocial support or skills-based training interventions that could help HCWs who care for PLHIV. Ideally, these should be integrated into a routine and ongoing training. However, in an overburdened health system, staff support is rarely seen as a priority, despite the important role that HCWs play in patients’ health behaviour and adherence.

### Strengths and limitations

This study provides valuable insight into the perspectives of HCWs toward patients who interrupt treatment which is limited in literature. The understanding of ART disengagement from the HCWs’ perspectives can help direct the development and implementation of interventions to support staff and change the environment around them that causes them to feel frustrated. It highlights the need to address HCWs’ negative attitudes and behaviours towards patients and encourage a more supportive and patient-centred approach to disengagement. Knowing which factors HCWs regard as justifiable could help when planning an intervention for patients as we will have an insight into which interventions the staff are likely to support; HCWs need to be on board to ensure that interventions are successful.

Although there was a large sample size and a diverse group of HCWs included, the study was conducted at one primary healthcare clinic which limits its applicability and relevance to other contexts and healthcare settings. This study was not able to show how HCWs feelings of empathy and anger play out during patient interactions. Future research is needed to understand how these perceptions impact people who disengage from care. The HCWs group is not homogeneous, therefore, for future research, there is a need to investigate how these different groups of HCWs could participate in addressing the issue of disengagement.

## Conclusion

HCW-patient relationships are complex, with tension amongst HCWs as they expressed both empathy and anger toward PLHIV patients who disengage from treatment. Patient engagement with ART involves many factors contributing to their retention and adherence. HCWs play an important role in patient empowerment and negative, punitive, or authoritarian attitudes can drive patients to disengage or reduce the likelihood that they re-engage with care. Although HCWs express empathy for patients, further work needs to be done to support staff to feel less overwhelmed by patients who disengage from treatment. This could include offering more psychosocial support for HCWs to address the attitudes and behaviours they may portray toward patients, as well as through capacitating HCWs to better support patients who disengage. Recognising that HCWs are gatekeepers to healthcare services, future interventions need to be designed to support both patients as well as the HCWs who care for them to improve ART engagement long term.

## Supplementary Information

**Additional file 1.**

## Data Availability

The data used in this study are stored by MSF and may be made available upon request from the corresponding author.

## Abbreviations

ABC: Actual event Beliefs and Consequences
ART: Antiretroviral therapy
CBT: Cognitive Behavioural Therapy
HCWs: Healthcare workers
HIV: Human Immunodeficiency Virus
MSF: Médecins Sans Frontières
PLHIV: People living with HIV
UNAIDS: Joint United Nations Programme on HIV and AIDS
WHO: World Health Organization

## Footnotes

1 ROTF = Risk of Treatment Failure; a differentiated service delivery model implemented at all primary care clinics in the Western Cape Province to support people who have a high VL (https://www.msf.org.za/sites/default/files/risk_of_treatment_failure_mentorship_toolkit_v3.pdf)
