# Supplementary material for: Between empathy and anger: healthcare workers’ perspectives on patient disengagement from antiretroviral treatment in Khayelitsha, South Africa - a qualitative study
Source: BMC Prim Care. 2023 Jan 26;24:34. doi: 10.1186/s12875-022-01957-8 (PMC9878968; doi:10.1186/s12875-022-01957-8)
Supplement: Supplementary file 1 — Additional file 1. [file 12875_2022_1957_MOESM1_ESM.docx]

Appendix A

Study number:

**State: date/ time/ location/ interview number**

1. Icebreaker to fill in the table of information: Firstly, can you tell me about yourself and your role here in the clinic?

| Age: |
| --- |
| Job title: doctor/nurse/counselor/navigator/pharmacist/pharmacy assistant/other  How long have you worked at this facility? (months/years)  How long have you worked in HIV? (months/years)  Affiliation: Department of Health/ NGO |

**We are going to talk about patients who disengage from HIV services: either missing appointments or becoming completely lost to follow up.**

1. What is ‘ disengagement’ from HIV care
   1. *What counts as disengagement/implications for patient health and resistance/ impact on patient care*

*Use the opportunity to clarify that we are talking about patients who have either completely disengaged from treatment (stopped taking their treatment and coming to the clinic completely) or who are intermittently engaged (missing appointments intermittently, missing viral load blood draws, missing pharmacy pick ups or have multiple high viral loads)*

1. Can you think of a specific patient who disengaged from HIV care? Who were they and what happened?
2. Which patients in your opinion are more likely to disengage from HIV care?
   1. *Common traits, socio-demographics, sex, personality*
3. How difficult do you think it is to take ARVs every day?
4. Why do patients disengage from HIV care?
   1. *Prioritization/ taking care seriously/responsibility*
   2. *Patient vs health system*
   3. *Specific reasons: side effects/ logistics/ social support/alcohol/ substance use/ treatment fatigue/violence.*
5. Are there any “good” or “understandable” reasons to disengage from care? Why?
   1. *Explore: side effects/ logistics/ social support/alcohol/ substance use/ treatment fatigue/violence*
6. Are there any “bad” or “unjustified” reasons to disengage from care? Why?
   1. *Explore: side effects/ logistics/ social support/alcohol/ substance use/ treatment fatigue/violence*
7. What is your opinion of patients who have disengaged from care or who intermittently engage with services?
   1. *Fault/ taking treatment seriously/ responsibility/ prioritisation/ Can this behavior of disengagement be described as normal?*
8. Who is to blame if a patient disengages from care?
   1. *Patient themselves/ circumstances/ society/ health system/ healthcare workers/ nobody*

**Now we are going to talk about dealing with these patients in the clinic setting (tailor to job description of participant and their interaction with patients)**

1. Can you think of a time when a patient reengaged in care? Who were they and what happened? What did you say to them? How did you feel when they reengaged
   1. *Impacts on your time, referrals, and prescriptions*
2. Describe what you think will happen if you tell the patient that interrupting treatment is normal?
3. Describe your role and responsibility with patients who have disengaged from care?
4. How do you feel when you have to manage/ deal with a patient who is returning to care?
   1. *What emotions do you feel?*
   2. *Worthy of the extra effort?*
5. Do you feel able to manage patients who disengage easily?
6. Explain what you find difficult about managing/ dealing with these patients?
   1. *Time-consuming/ complicated to manage/ emotionally draining/ill-equipped to manage/ lack of referral options for patient.*

**Now we are going to talk about disengagement more broadly**

1. Do you think disengagement is a problem that warrants focus/resources? Why? Which patients should get the highest priority?
   1. *Worthy of extra resources*
2. For patients who disengage from treatment and services, do you think it is possible for healthcare workers to change the way these patients behave? Why?
   1. *Futility vs worthy of additional effort*
3. What do you need to be able to manage these patients well?
